# A Characterization of Codimension One Collapse Under Bounded Curvature and Diameter

**DOI:** 10.1007/s12220-017-9930-0

**Published:** 2017-10-03

**Authors:** Saskia Roos

**Affiliations:** Max-Planck-Institut für Mathematik, Vivatsgasse 7, 53111 Bonn, Germany

**Keywords:** Collapsing, Gromov–Hausdorff convergence, Riemannian submersions, 53B20, 53C20, 58A30

## Abstract

Let $\mathcal {M}(n,D)$ be the space of closed *n*-dimensional Riemannian manifolds (*M*, *g*) with ${{\mathrm{diam}}}(M) \le D$ and $\vert \sec ^M \vert \le 1$. In this paper we consider sequences $(M_i,g_i)$ in $\mathcal {M}(n,D)$ converging in the Gromov–Hausdorff topology to a compact metric space *Y*. We show, on the one hand, that the limit space of this sequence has at most codimension one if there is a positive number *r* such that the quotient $\frac{{{\mathrm{vol}}}(B^{M_i}_r(x))}{{{\mathrm{inj}}}^{M_i}(x)}$ can be uniformly bounded from below by a positive constant *C*(*n*, *r*, *Y*) for all points $x \in M_i$. On the other hand, we show that if the limit space has at most codimension one then for all positive *r* there is a positive constant *C*(*n*, *r*, *Y*) bounding the quotient $\frac{{{\mathrm{vol}}}(B^{M_i}_r(x))}{{{\mathrm{inj}}}^{M_i}(x)}$ uniformly from below for all $x \in M_i$. As a conclusion, we derive a uniform lower bound on the volume and a bound on the essential supremum of the sectional curvature for the closure of the space consisting of all manifolds in $\mathcal {M}(n,D)$ with $C \le \frac{{{\mathrm{vol}}}(M)}{{{\mathrm{inj}}}(M)}$.

## Introduction

Let $\mathcal {M}(n,D)$ be the space of isometry classes of closed *n*-dimensional Riemannian manifolds (*M*, *g*) with ${{\mathrm{diam}}}(M) \le D$ and $\vert \sec ^M \vert \le 1$. Due to Gromov, [11], it is known that this space is precompact with respect to the Gromov–Hausdorff metric $d_{\text {GH}}$. A $d_{\text {GH}}$-convergent sequence $(M_i, g_i)_{i \in \mathbb {N}}$ in $\mathcal {M}(n,D)$ is said to *collapse* if its limit space is of lower dimension.

The first nontrivial example of collapse was discovered and carried out by Marcel Berger in about 1962. He considered the Hopf fibration $\mathbb {S}^1 \rightarrow \mathbb {S}^3 \rightarrow \mathbb {S}^2(\frac{1}{2})$ and rescaled the metric tangent to the fibers by $\varepsilon >0$ while keeping the original metric in the directions orthogonal to the fibers fixed. As $\varepsilon \rightarrow 0$ the sectional curvature remains bounded while the injectivity radius converges uniformly to 0 at each point. Furthermore, $\mathbb {S}^3$ resembles more and more a two-sphere with constant sectional curvature 4 as $\varepsilon \rightarrow 0$.

A cornerstone for the theory of collapse under bounded curvature is Gromov’s characterization of almost flat manifolds [10]. For example, Fukaya’s fibration theorems [6, 8] can be understood as a parametrized version of [10]. In [7], Fukaya applied these fibration theorems to the sequence of frame bundles of a collapsing sequence in $\mathcal {M}(n,D)$ and derived a description of the boundary of $\mathcal {M}(n,D)$ (see [7, Theorem 10.1]). Cheeger et al. proved a simultaneously equivariant and parametrized version of this result in [2].

It is well known that in general the elements in the boundary of $\mathcal {M}(n,D)$ have singularities. Fukaya showed in [7] that the Hausdorff dimension of elements in the boundary of $\mathcal {M}(n,D)$ is an integer. If the limit space of a collapsing sequence in $\mathcal {M}(n,D)$ has codimension one, Fukaya proved that it has to be a Riemannian orbifold with an induced $C^{1, \alpha }$-metric, [9, Proposition 11.5]. This motivates the main result of this paper which provides the following equivalent characterizations on sequences in $\mathcal {M}(n,D)$ that drop at most one dimension in the limit.

### Theorem 1.1

Let $(M_i , g_i)_{i \in \mathbb {N}}$ be a sequence in $\mathcal {M}(n,D)$ converging to a compact metric space (*Y*, *d*) in the Gromov–Hausdorff topology. Then the following are equivalent

(i) $$\dim _{\mathrm {Haus}}(Y) \ge (n-1)$$,
(ii) for all $r >0$ there is a positive constant *C*(*n*, *r*, *Y*) such that
  1.1 $$\begin{aligned} C \le \frac{{{\mathrm{vol}}}(B^{M_i}_r(x))}{{{\mathrm{inj}}}^{M_i}(x)} \end{aligned}$$
  holds for all $x \in M_i$ and all $i \in \mathbb {N}$,
(iii) for some $r >0$ there is a positive constant *C*(*n*, *r*, *Y*) such that (1.1) holds for all $x \in M_i$ and all $i \in \mathbb {N}$.

The idea behind Theorem 1.1 is the following illustrative observation. Let $(M_i, g_i)_{i \in \mathbb {N}}$ be a collapsing sequence in $\mathcal {M}(n,D)$. Then the *r*-balls around a sequence of points $x_i \in M_i$ contain all collapsing directions, while the injectivity radii at the points $x_i$ only represent the fastest scale of collapsing. Now, if the collapse has codimension one, it happens on the scale of the injectivity radius. Hence, the volume of the balls $B^{M_i}_r(x_i)$ and the injectivity radii ${{\mathrm{inj}}}^{M_i}(x_i)$ converge to 0 at the same rate. Therefore, the ratio (1.1) can be uniformly bounded from below. However, if the collapse has codimension larger than two, the injectivity radius only represents the fastest scale of collapsing. Thus, the volume of the balls $B^{M_i}_r(x_i)$ converges on a larger scale to 0 than the injectivity radii ${{\mathrm{inj}}}^{M_i}(x_i)$. Consequently, their quotients converge to 0.

### Example 1.2

Consider the following sequence of flat tori $$(T_i :=\mathbb {S}^1 \times \frac{1}{i} \mathbb {S}^1 , g_i)_{i \in \mathbb {N}}$$. Here $$g_i$$ is the product metric on the product of circles of radii 1 and $$\frac{1}{i}$$. This sequence collapses to $$\mathbb {S}^1$$ which is of codimension one. As $${{\mathrm{inj}}}^{T_i}(x) \equiv \frac{\pi }{i}$$, it follows that for any $$ r >0$$ and $$x \in T_i$$, we have that $${{\mathrm{vol}}}(B^{T_i}_r(x)) \approx 2 \min \lbrace r, \pi \rbrace \cdot \frac{2\pi }{i}$$ as *i* tends to infinity. Therefore, we derive for $$r \le \pi $$ that

$$\begin{aligned} \lim _{i \rightarrow \infty } \frac{{{\mathrm{vol}}}(B^{T_i}_r(x))}{{{\mathrm{inj}}}^{T_i}(x)} = \lim _{i \rightarrow \infty } \frac{4 r \frac{\pi }{i}}{\frac{\pi }{i}} = 4 r > 0. \end{aligned}$$

Thus, this quotient can be uniformly bounded by a positive constant *C*, as stated in Theorem 1.1.

### Example 1.3

Similarly to the previous example we consider the sequence of flat tori $(T_j :=\frac{1}{j^2} \mathbb {S}^1 \times \frac{1}{j} \mathbb {S}^1, g_j)_{j \in \mathbb {N}}$. This sequence collapses to a point. No matter how small we choose $r > 0$ there exists some $J \in \mathbb {N}$ such that ${{\mathrm{vol}}}(B^{T_j}_r(x)) = \frac{2 \pi }{j^2} \cdot \frac{2\pi }{j}$ for any $j > J$. As ${{\mathrm{inj}}}^{T_j}(x) \equiv \frac{\pi }{j^2}$, the same considerations as before lead to

$$\begin{aligned} \lim _{j \rightarrow \infty } \frac{{{\mathrm{vol}}}(B^{T_j}_r(x))}{{{\mathrm{inj}}}^{T_j}(x)} = \lim _{j \rightarrow \infty } \frac{4 \pi }{j} =0. \end{aligned}$$

Therefore, we cannot find a uniform positive lower bound for this quotient.

The proof of Theorem 1.1 requires the improved version of Fukaya’s fibration theorems derived in [2]. First, we show that, in order to prove Theorem 1.1, it is enough to restrict to sequences of manifolds with invariant metrics, as introduced in [2]. Then we prove that (i) implies (ii) by constructing a lower bound as required in (1.1) for any given $r>0$. As the implication from (ii) to (iii) is trivial it remains to show that (iii) implies (i). This direction will be proved by contradiction. We will bound the volume of the ball in the manifold, up to a constant, by the injectivity radius and the diameter of the collapsing fibers. It remains to bound the injectivity radius of the fibers from above by the injectivity radius of the manifold in the related points. This is done by modifying the results of [19] for bounded Riemannian submersions. In the end, we show that the constructed upper bound on the quotient converges to 0, giving a contradiction.

The paper is organized as follows. In Sect. 2 we recall the theory of collapsing sequences in $\mathcal {M}(n,D)$ developed by Cheeger, Fukaya, and Gromov. In particular, we recall that each sufficiently collapsed manifold is a singular fibration with infranil fibers.

Section 3 deals with bounded Riemannian submersions $\eta : M \rightarrow Y$, i.e., Riemannian submersions where the norm of the fundamental tensors *A* and *T* is bounded by positive constants $C_A$ resp. $C_T$. There we modify the results of [19] to obtain the following upper bound on the injectivity radius of the fibers $F_p :=\eta ^{-1}(\lbrace p \rbrace )$, $p \in Y$.

### Proposition 1.4

Let $\eta : M^{n+k} \rightarrow Y^{n}$ be a bounded Riemannian submersion with $\vert \sec ^M \vert \le K$ for some $K > 0$. Assume further that ${{\mathrm{inj}}}^M(x) < \min \bigg \lbrace \frac{\pi }{\sqrt{K + 3C_A^2}} , \; \frac{1}{4}{{\mathrm{inj}}}^Y(p) \bigg \rbrace$ for some $x \in F_p$. Then

$$\begin{aligned} {{\mathrm{inj}}}(F_p) \le \Big (1 + \tau ({{\mathrm{inj}}}^M(x) \vert C_A, C_T, k, K) \Big ) \cdot {{\mathrm{inj}}}^M(x). \end{aligned}$$

Here $\tau (\varepsilon \vert a_1 , \ldots , a_l)$ denotes a positive continuous function such that $\lim _{\varepsilon \rightarrow 0} \tau (\varepsilon \vert a_1 , \ldots , a_l) =0$  for  fixed $a_1 , \ldots , a_l$. The explicit expression of the constant $\Big (1 + \tau ({{\mathrm{inj}}}^M(x) \vert C_A, C_T, k, K) \Big )$ is given in the proof.

In Sect. 4 we prove Theorem 1.1 using the strategy explained above. In conclusion, we define the space $\mathcal {M}(n,D,C)$ consisting of all manifolds in $\mathcal {M}(n,D)$ satisfying $C \le \frac{{{\mathrm{vol}}}(M)}{{{\mathrm{inj}}}(M)}$. We show that there is a uniform bound on the essential supremum of the sectional curvature and a uniform lower bound on the volume for all $(n-1)$-dimensional metric spaces *Y* in the closure of $\mathcal {M}(n,D,C)$.

## Collapsing Theory for Bounded Curvature and Diameter

In this section we recall the relevant theorems about convergence and collapsing in $\mathcal {M}(n,D)$. From this point on, we use the following notation: $\tau (\varepsilon \vert a_1, \ldots , a_l)$ denotes a positive continuous function depending on $\varepsilon$ and $a_1, \ldots , a_l$ such that $\lim _{\varepsilon \rightarrow 0} \tau (\varepsilon \vert a_1, \ldots , a_l) = 0$ for any fixed $a_1, \ldots , a_l$.

In [11, Theorem 5.3], Gromov proved that $\mathcal {M}(n,D)$ is precompact in the Gromov–Hausdorff topology. Furthermore, for any fixed positive $i_0$ the subspace

$$\begin{aligned} \mathcal {M}(n,D,i_0) :=\lbrace (M,g) \in \mathcal {M}(n,D)\vert {{\mathrm{inj}}}^M > i_0 \rbrace \end{aligned}$$

is precompact in the $C^{1, \alpha }$-topology (see [5, 16]).

Without the uniform lower bound on the injectivity radius, a sequence $(M_i, g_i)_{i \in \mathbb {N}}$ in $\mathcal {M}(n,D)$ might *collapse* to a metric space of lower Hausdorff dimension. In [7], Fukaya studied collapsing sequences in $\mathcal {M}(n,D)$ considering the corresponding sequence of frame bundles $FM_i$.

### Theorem 2.1

(Fukaya) Let $(M_i, g_i)_{i \in \mathbb {N}}$ be a sequence in $\mathcal {M}(n,D)$ converging with respect to the Gromov–Hausdorff metric to a compact metric space *Y*. There exists a positive $\varepsilon :=\varepsilon (n,D)$ such that, for all *i* with $d_{\text {GH}}(M_i,Y) \le \varepsilon$, there is a map $\eta _i: M_i \rightarrow Y$, and a manifold $\tilde{Y}$ on which *O*(*n*) acts isometrically, and an *O*(*n*)-equivariant map $\tilde{\eta }_i:FM_i \rightarrow \tilde{Y}$ such that2.1commutes, and

(i) $\tilde{Y}$ is a Riemannian manifold with $C^{1, \alpha }$-metric tensors,
(ii) $\tilde{\eta }_i$ is a fiber bundle with affine structure group and infranil fibers,
(iii) $\tilde{\eta }_i$ is an almost Riemannian submersion, i.e., if $X \in T_x FM_i$ is perpendicular to the fibers of $\tilde{\eta }_i$, then
  $$\begin{aligned} e^{- \tau (d_{\text {GH}}(M_i,Y) \vert n,D)}< \frac{\Vert d\tilde{\eta }_i(X) \Vert }{\Vert X \Vert } < e^{\tau (d_{\text {GH}}(M_i,Y) \vert n, D)}, \end{aligned}$$
(iv) $M_i$ and *Y* are isometric to $FM_i / O(n)$ and $\tilde{Y}/O(n)$, respectively,
(v) for each $p \in Y$ the groups $G_{\tilde{p}} = \lbrace g \in O(n) \vert g(\tilde{p}) = \tilde{p})$ for $\tilde{p} \in \pi ^{-1}(p)$ are isomorphic to each other. We set $G_{p} :=G_{\tilde{p}}$ for some fixed $\tilde{p}\in \pi ^{-1}(p)$.

We henceforth use the notation introduced in this theorem repeatedly.

Another approach to collapse under bounded curvature was carried out by Cheeger and Gromov [3, 4]. They generalized local group actions and introduced an action of a sheaf of groups. In particular, they considered actions of sheaves of tori with additional regularity conditions. This defines the so-called *F*-structure (“*F*” stands for *flat*). Cheeger and Gromov proved that each sufficiently collapsed complete Riemannian manifold admits an *F*-structure of positive rank. This approach does not require an upper bound on the diameter of the manifold.

Combining these two approaches, Cheeger et al. introduced in [2] a nilpotent structure (*N*-structure) and showed its existence on each sufficiently collapsed part of a complete Riemannian manifold. Roughly, if *M* is sufficiently collapsed, its frame bundle *FM* is the total space of a fibration with infranil fibers and affine structure group. Thus, there is a sheaf on *FM* whose local sections are given by local right invariant vector fields on the fiber.

A further main result of their article [2] is the existence of *invariant metrics* on manifolds admitting an *N*-structure. These metrics are invariant in the sense that the local sections of the sheaf on the frame bundle are given by local Killing fields.

To obtain such a metric they first applied the following theorem due to Abresch [1] to obtain uniform bounds on the derivatives of the curvature (see also [18]).

### Theorem 2.2

(Abresch) For any $\varepsilon >0$ and $n \in \mathbb {N}$ there is a smoothing operator $S_{\varepsilon }$ such that on any complete Riemannian manifold (*M*, *g*) with $\vert \sec ^g \vert \le 1$ the metric $\tilde{g} :=S_{\varepsilon }(g)$ satisfies

(i) $$e^{-\varepsilon }g< \tilde{g} < e^{\varepsilon }g$$,
(ii) $$\vert \nabla - \tilde{\nabla } \vert < \varepsilon $$,
(iii) $\vert \tilde{\nabla }^j \tilde{R} \vert < A_j(n,\varepsilon )$ for all $j \ge 0$.

In addition, Rong showed that, for sufficiently small $\varepsilon$, we have the following bounds for the sectional curvature of $S_{\varepsilon }(g)$, c.f. [17, Proposition 2.5].

### Proposition 2.3

(Rong) There is a constant $\delta > 0$ such that for any complete Riemannian manifold (*M*, *g*) with $\vert \sec ^g \vert \le 1$ and any $0 \le \varepsilon \le \delta$, there is a positive constant *C*(*n*) such that the metric $\tilde{g} :=S_{\varepsilon }(g)$ satisfies

$$\begin{aligned} \min \sec ^{\tilde{g}} -\,C(n)\varepsilon \le \sec ^g \le \max \sec ^{\tilde{g}} + \,C(n) \varepsilon . \end{aligned}$$

At this point, we introduce the following notion: A Riemannian manifold (*M*, *g*) is called *A*-*regular* if there is a sequence $A =(A_j)_{j \in \mathbb {N}}$ of nonnegative numbers such that $\vert \nabla ^j R \vert \le A_j$ for all $j \ge 0$. Furthermore, *C*(*A*) will denote a constant depending on $A_j$ for finitely many $j \ge 0$.

Assuming the manifold to be *A*-regular, Cheeger, Fukaya, and Gromov proved the existence of invariant metrics, compare [2, Sect. 7, Sect. 8].

### Theorem 2.4

(Cheeger–Fukaya–Gromov) Let $(M,g) \in \mathcal {M}(n,D)$ be an *A*-regular Riemannian manifold such that there is a lower dimensional metric space *Y* with $d_{\text {GH}}(M,Y) \le \varepsilon (n,D)$, where $\varepsilon (n,D)$ is the constant in Theorem 2.1. Then there is an invariant metric $\tilde{g}$ such that

$$\begin{aligned} \vert \nabla ^j(g - \tilde{g}) \vert \le c(n,A,j) d_{\text {GH}}(M,Y). \end{aligned}$$

Here $\varepsilon (n,D)$ is the constant from Theorem 2.1. Furthermore, the map $\tilde{\eta }: FM \rightarrow \tilde{Y}$ is a Riemannian submersion with respect to the metric induced by $\tilde{g}$ such that the second fundamental form of the fibers is bounded by a positive constant *C*(*n*, *A*).

## The Injectivity Radius of the Fiber

The goal of this section is to prove Proposition 1.4. Therefore, consider a Riemannian submersion $\eta : M \rightarrow Y$. Henceforth, denotes the fiber over $p \in Y$ by $F_p :=\eta ^{-1}(\lbrace p \rbrace )$ and $k :=\dim (F_p).$

Recall that a Riemannian submersion is *bounded* if the fundamental tensors *A* and *T* are bounded in norm by positive constants $C_A$ resp. $C_T$.

The main ingredient of the proof of Proposition 1.4 is a homotopy with fixed endpoints between a curve $\gamma$ with endpoints in a fiber $F_p$ and a curve $\tilde{\gamma }$ lying completely in the fiber $F_p$ such that the length of $\tilde{\gamma }$ is bounded from above linear in terms of $l(\gamma )$. Such a homotopy was constructed in the proof of Theorem 3.1 in [19].

### Proposition 3.1

(Tapp) Let $\eta : M \rightarrow Y$ be a bounded Riemannian submersion with *Y* being compact and simply connected. Then there exists a positive constant $C :=C(Y,k,C_T, C_A)$ such that any curve $\gamma$ in *M* with $\eta \circ \gamma$ being a contractible loop is homotopic to a curve $\tilde{\gamma }$ in the fiber $F_p$, $p = \eta \circ \gamma (0)$, satisfying

$$\begin{aligned} l(\tilde{\gamma }) \le C \, l(\gamma ). \end{aligned}$$

Comparing the assumptions of Proposition 3.1 with those of Proposition 1.4, there are a few differences. First, in Proposition 3.1, Tapp requires *Y* to be compact and simply connected. These assumptions are needed to guarantee that for any loop $\alpha : [0,1] \rightarrow Y$ there is a nullhomotopy $H:[0,1] \times [0,1] \rightarrow Y$, i.e., $H(1,t) = \alpha (t)$, $H(0,t) = \alpha (0)$, and $H(s,0)= H(s,1)= \alpha (0)$ for all $s \in [0,1]$, whose derivatives are uniformly bounded, c.f. [19, Lemma 7.2].

Going back to the statement of Proposition 1.4, the assumptions therein imply that the considered noncontractible geodesic loop $\gamma$ based at $x \in F_p$ has length $l(\gamma ) = 2 {{\mathrm{inj}}}^M(x) < \frac{1}{2} {{\mathrm{inj}}}^Y(p)$. Thus, the loop $\eta \circ \gamma$ is contractible in *Y*. Furthermore, by assuming a bound on the sectional curvature of *Y* there is a nullhomotopy for curves with length less or equal $\frac{1}{2}{{\mathrm{inj}}}^Y(p)$ whose derivatives can be bounded as follows:

### Lemma 3.2

Let *Y* be a Riemannian manifold with $- \,\lambda ^2 \le \sec ^Y \le \Lambda ^2$ for some $\lambda , \Lambda > 0$. Furthermore, let $\alpha :[0,1] \rightarrow Y$ be a loop in *Y* based at *p* and $l(\alpha ) < \min \lbrace \frac{2\pi }{\Lambda } , \frac{1}{2} {{\mathrm{inj}}}^Y(p) \rbrace$. Then there is a piecewise smooth nullhomotopy $H: [0,1] \times [0,1] \rightarrow Y$, i.e., $H(0,t)= p$ and $H(1,t) = \alpha (t)$ and $H(s,0) = H(s,1)=p$ for all $s \in [0,1]$, such that

$$\begin{aligned} \left| \frac{\partial }{\partial t} H \right|&\le \frac{\Lambda }{\lambda } \cdot \frac{\sinh \left( \lambda \; \frac{l(\alpha )}{2}\right) }{\sin \left( \Lambda \; \frac{l(\alpha )}{2}\right) } \cdot l(\alpha ) , \\ \left| \frac{\partial }{\partial s} H \right|&\le \frac{\sinh \left( \lambda \; \frac{l(\alpha )}{2}\right) }{\lambda \frac{l(\alpha )}{2}} \cdot \frac{l(\alpha )}{2} \end{aligned}$$

for all *s*, $$t \in [0,1]$$.

### Proof

Let $\alpha$ be parametrized proportional to arclength. Since $\alpha$ satisfies $l(\alpha ) < \frac{1}{2} {{\mathrm{inj}}}^Y(p),$ it lifts to a loop $\tilde{\alpha } :=\exp _p^{-1} \circ \, \alpha$ in $T_pY$.

Define $\tilde{H}(s,t) :=s \cdot \tilde{\alpha }(t)$ with *s*, $t \in [0,1]$. Clearly, we have that

$$\begin{aligned} \left| \frac{\partial }{\partial s} \tilde{H} \right| = \vert \tilde{\alpha } \vert \le \frac{l(\alpha )}{2}. \end{aligned}$$

To estimate $\left| \frac{\partial }{\partial t} \tilde{H} \right|$, we first observe that, as $- \lambda ^2 \le \sec ^Y \le \Lambda ^2$, it follows that

$$\begin{aligned} \frac{\sin (\Lambda \vert v \vert )}{\Lambda \vert v \vert } \vert w \vert \le \vert (\mathrm {D}_{v} \exp _p) (w) \vert \le \frac{\sinh (\lambda \vert v \vert )}{\lambda \vert v \vert } \vert w \vert \end{aligned}$$

for all $v \in T_pY$ with $\vert v \vert < \frac{\pi }{\lambda }$ and $w \in T_v T_p Y$, see, e.g., [14, Corollary 4.6.1]. Therefore, we obtain for $q \in B_{\frac{1}{4} {{\mathrm{inj}}}^Y(p)}(p)$ and $u \in T_q Y$ that

$$\begin{aligned} \vert ( \mathrm {D}_q \exp _p^{-1} ) u \vert \le \frac{\Lambda \vert \exp ^{-1} (q) \vert }{\sin ( \Lambda \vert \exp ^{-1} (q) \vert )} \vert u \vert . \end{aligned}$$

Thus,

$$\begin{aligned} \left| \frac{\partial }{\partial t} \tilde{H} \right| \le \left| \frac{\partial }{\partial t} \tilde{\alpha } \right| \le \frac{\Lambda \vert \tilde{\alpha } \vert }{\sin (\Lambda \vert \tilde{\alpha } \vert )} \left| \frac{\partial }{\partial t} \alpha \right| \le \frac{\Lambda \frac{l(\alpha )}{2}}{\sin \left( \Lambda \; \frac{l(\alpha )}{2}\right) } \cdot l(\alpha ). \end{aligned}$$

By construction $H :=\exp _p (\tilde{H})$ is a piecewise smooth nullhomotopy of $\alpha$ in *Y* such that

$$\begin{aligned} \left| \frac{\partial }{\partial s} H \right| \le \frac{\sinh (\lambda \vert \tilde{H}(s,t) \vert )}{\lambda \vert \tilde{H}(s,t) \vert } \, \left| \frac{\partial }{\partial s} \tilde{H} \right| \le \frac{\sinh \left( \lambda \; \frac{l(\alpha )}{2}\right) }{\lambda \frac{l(\alpha )}{2}} \, \cdot \frac{l(\alpha )}{2}. \end{aligned}$$

The corresponding bound on $\left| \frac{\partial }{\partial t} H \right|$ is derived similarly. $\square$

The next corollary follows immediately by adjusting the bounds on the derivative of the exponential map.

### Corollary 3.3

Let *Y* be a Riemannian manifold with $- \lambda ^2 \le \sec ^Y \le - \Lambda ^2$ for some $\lambda \ge \Lambda \ge 0$. Furthermore, let $\alpha : [0,1] \rightarrow Y$ be a loop in *Y* based at *p* and $l(\alpha ) < \frac{1}{2} {{\mathrm{inj}}}^Y(p)$. Then there is a piecewise smooth nullhomotopy $H:[0,1] \times [0,1] \rightarrow Y$, as before, such that

$$\begin{aligned} \left| \frac{\partial }{\partial t} H \right|&\le \frac{\Lambda }{\lambda } \cdot \frac{\sinh \left( \lambda \; \frac{l(\alpha )}{2}\right) }{\sinh \left( \Lambda \; \frac{l(\alpha )}{2}\right) } \cdot l(\alpha ) , \\ \left| \frac{\partial }{\partial s} H \right|&\le \frac{\sinh \left( \lambda \; \frac{l(\alpha )}{2}\right) }{\lambda \frac{l(\alpha )}{2}} \cdot \frac{l(\alpha )}{2}. \end{aligned}$$

In the case of $\Lambda =0$, we set $\frac{\sinh (\Lambda )}{\Lambda } = 1$.

Next, we prove Proposition 1.4. Therein we keep carefully track of the dependence of the constants on ${{\mathrm{inj}}}^M(x)$ because this is the quantity going to 0 in a collapsing sequence, while the other quantities will be uniformly bounded.

### Proof of Proposition 1.4

As ${{\mathrm{inj}}}^M(x) < \frac{\pi }{\sqrt{K}}$ there is a noncontractible geodesic loop $\gamma$ based at *x* such that $l(\gamma ) = 2 {{\mathrm{inj}}}^M(x)$. As ${{\mathrm{inj}}}^M(x) < \frac{1}{4} {{\mathrm{inj}}}^Y(p)$, the composition $\eta \circ \gamma$ is contractible in *Y*. By Proposition 3.1, $\gamma$ is homotopic to a noncontractible loop $\tilde{\gamma }$ in the fiber $F_p$ such that $l( \tilde{\gamma } ) \le C \cdot l(\gamma )$ for a positive constant $C :=C(Y,k,C_A,C_T)$. Thus,

$$\begin{aligned} 2 {{\mathrm{inj}}}^{F_p} \le l(\tilde{\gamma }) \le C \cdot l(\gamma ) = C \cdot 2 {{\mathrm{inj}}}^M(x). \end{aligned}$$

We claim that $C = \tau ( l(\gamma ) \vert C_A, C_T, k, K )$. The proof consists of a careful study of the constant *C*, following the proof of [19, Theorem 3.1]. In this proof, Tapp modifies the path $\gamma$ such that it is a concatenation of paths with endpoints in the fiber whose length is not larger than $( 2 {{\mathrm{diam}}}(Y) + 1)$. As $l(\gamma ) < (2 {{\mathrm{diam}}}(Y) + 1)$ already holds, by assumption we do not need this modification.

Set $\alpha (t) :=\eta \circ \gamma$. The vertical curve $\tilde{\gamma }$ is the concatenation of the paths $\beta _1$ and $\beta _2$. The first path $\beta _1$ goes from *x* to $z :=h^{\alpha }(x)$, where $h^{\alpha }: F_p \rightarrow F_p$ is the holonomy diffeomorphism associated to $\alpha$ and the path $\beta _2$ connects *z* with *x* again. Hence,

$$\begin{aligned} l(\tilde{\gamma }) \le l(\beta _1) + l(\beta _2) \le P \cdot l(\gamma ) + L \cdot l(\gamma ) = C l(\gamma ) \end{aligned}$$

for some explicit positive constants *P* and *L*, compare with [19, p. 645]. We will study these constants *P* and *L* in detail.

First we consider the inequality $l(\beta _1) \le P \cdot l(\gamma )$. The constant *P* is a bound on the derivative of the function $l \mapsto \rho _l (1)$ between $l = 0$ and $l = l(\gamma )$, where $\rho _{l(\gamma )} (t)$ is the solution to the differential equation

3.1 $$\begin{aligned} \begin{aligned} (\rho _{l(\gamma )})^{\prime }(t)&= kC_A Q_s Q_t l(\gamma ) ( 1 + 4^k k!) + k Q_t l ( C_T + 4^k k! C_A) \rho _l(t) \ ;\\ \rho _{l(\gamma )}(0)&= 0. \end{aligned} \end{aligned}$$

The constants $Q_t$ and $Q_s$ are the bounds on the nullhomotopy *H* of the path $\alpha (t)$ in *Y*, i.e., $\left| \frac{\partial }{\partial t} H \right| \le Q_t l(\gamma )$ and $\left| \frac{\partial }{\partial s} H \right| \le Q_s$ uniformly.

As, by Gray-O’Neill’s formula, $-K \le \sec ^Y \le ( K + 3C_A^2 )$ and, by assumption, $l(\alpha ) \le l(\gamma ) < \min \bigg \lbrace \frac{2 \pi }{\sqrt{K + 3C_A^2}}, \frac{1}{2} {{\mathrm{inj}}}^Y(p)\bigg \rbrace$, we apply Lemma 3.2 and obtain

$$\begin{aligned} Q_t&= \frac{\sqrt{K + 3C_A^2}}{\sqrt{K}} \cdot \frac{\sinh \left( \sqrt{K} \frac{l(\gamma )}{2} \right) }{\sin \left( \sqrt{K + 3 C_A^2} \frac{l(\gamma )}{2}\right) } , \\ Q_s&= \frac{\sinh \left( \sqrt{K} \frac{l(\gamma )}{2}\right) }{\sqrt{K} \frac{l(\gamma )}{2}} \cdot \frac{l(\gamma )}{2} =:\tilde{Q}_s l(\gamma ). \end{aligned}$$

Note that for any loop $\bar{\alpha }$ in *Y* of length less or equal than $l(\gamma )$ the corresponding nullhomotopy $\bar{H}$ of $\bar{\alpha }$ satisfies the bounds $\left| \frac{\partial }{\partial t} \bar{H} \right| \le Q_t l(\bar{\alpha })$ and $\left| \frac{\partial }{\partial s} \bar{H} \right| \le \tilde{Q}_s l(\bar{\alpha }).$

Thus, in our case, the differential equation (3.1) reads as

$$\begin{aligned} (\rho _l)^{\prime }(t)&= k C_A \tilde{Q}_s Q_t l^2 (1 + 4^k k!) + k Q_t l (C_T + 4^k k! C_A) \rho _l (t) \ ;\\ \rho _l(0)&= 0, \end{aligned}$$

for $0 \le l \le l(\gamma )$, compare [19, Lemma 3.3]. For simplicity, set

$$\begin{aligned} G_1&:=k C_A \tilde{Q}_s Q_t (1 + 4^k k!), \\ G_2&:=k Q_t (C_T + 4^k k! C_A). \end{aligned}$$

Using the variation of constants, we conclude

$$\begin{aligned} \rho _l(1) = \frac{G_1}{G_2} l \big ( e^{G_2 l} -1 \big ). \end{aligned}$$

Therefore,

3.2 $$\begin{aligned} \frac{\mathrm {d}}{\mathrm {d} l} \rho _l(1)&= \frac{G_1}{G_2}\big ( e^{G_2 l} -1 \big ) + G_1 l e^{G_2 l}\end{aligned}$$

3.3 $$\begin{aligned}&\le \frac{G_1}{G_2}\big ( e^{G_2 l(\gamma )} -1 \big ) + G_1 l(\gamma ) e^{G_2 l(\gamma )} =:P. \end{aligned}$$

It remains to check the behavior of the appearing quantities as $l(\gamma )$ becomes small. Since $Q_t$ and $\tilde{Q}_s$ are the only appearing quantities that depend on $l(\gamma )$, we first note that

$$\begin{aligned} \lim _{l(\gamma ) \rightarrow 0 } Q_t&= 1,\\ \lim _{l(\gamma ) \rightarrow 0} \tilde{Q}_s&= \frac{1}{2}. \end{aligned}$$

Therefore, we extract the quantities $Q_t$ and $\tilde{Q}_s$ in (3.2). This is done by considering each term separately. We observe that

$$\begin{aligned} \frac{G_1}{G_2}&= \frac{k C_A \tilde{Q}_s Q_t (1 + 4^k k!)}{k Q_t (C_T + 4^k k! C_A)} = \tilde{Q}_s \frac{C_A(1+4^k k!)}{C_T + 4^k k! C_A} =:\tilde{Q}_s \cdot C_1(C_A,C_T, k), \\ G_1 l(\gamma )&= k C_A \tilde{Q}_s Q_t (1 + 4^k k!) l(\gamma ) =:\tilde{Q}_s Q_t l(\gamma ) \cdot C_2 (C_A, k),\\ G_2 l(\gamma )&= k Q_t (C_T + 4^k k! C_A) l(\gamma ) =:Q_t l(\gamma ) \cdot C_3(C_A,C_T,k). \end{aligned}$$

As $l(\gamma )$ becomes small, we obtain

$$\begin{aligned} \lim _{l(\gamma ) \rightarrow 0} \frac{G_1}{G_2}&= \frac{1}{2} \cdot C_1(C_A, C_T, k),\\ \lim _{l(\gamma ) \rightarrow 0} G_1 l(\gamma )&= 0 \cdot C_2(C_A, k) = 0,\\ \lim _{l(\gamma ) \rightarrow 0} G_2 l(\gamma )&= 0 \cdot C_3(C_A,C_T,k) = 0. \end{aligned}$$

Summarizing these observations we conclude

$$\begin{aligned} \lim _{l(\gamma )\rightarrow 0} P&= \lim _{l(\gamma )\rightarrow 0} \frac{G_1}{G_2}\big ( e^{G_2 l} -1 \big ) + G_1 l e^{G_2 l}\\&\le \lim _{l(\gamma )\rightarrow 0} \frac{G_1}{G_2}\big ( e^{G_2 l(\gamma )} -1 \big ) + G_1 l(\gamma ) e^{G_2 l(\gamma )}\\&= \frac{1}{2}C_1(C_A,C_T,k) \big (e^0 -1 \big ) + 0 \cdot e^0 = 0. \end{aligned}$$

This shows that $P = \tau (l(\gamma ) \vert C_A, C_T, k, K)$.

Next, we consider the inequality $l(\beta _2) \le L \cdot l(\gamma )$. Here, *L* is the maximum of the Lipschitz constants of the holonomy diffeomorphism $h^{\alpha }$ associated to paths $\alpha$ in *Y*. Since $h^{\alpha }$ satisfies the Lipschitz constant $e^{C_T \cdot l(\alpha )}$ (c.f. [12, Lemma 4.2]) and $l(\alpha )$ is bounded from above by $l(\gamma )$, we conclude that

$$\begin{aligned} L = e^{C_T \cdot l(\gamma )} =1 + \tau (l(\gamma ) \vert C_T ). \end{aligned}$$

Together with $P = \tau (l(\gamma ) \vert C_A, C_T, k, K)$, this shows the claim.

Thus, we finally obtain

$$\begin{aligned} 2{{\mathrm{inj}}}^{F_p} \le l(\tilde{\gamma })&\le C \cdot l(\gamma ) \\&= \Big (1 + \tau ( l(\gamma ) \vert C_A, C_T, k, K) \Big ) \cdot l(\gamma ) \\&= \Big (1 + \tilde{\tau }( {{\mathrm{inj}}}^M(x) \vert C_A, C_T, k, K) \Big ) \cdot 2 {{\mathrm{inj}}}^M(x). \end{aligned}$$

$$\square $$

### Remark 3.4

If $- \,K \le \sec ^M \le - \,\kappa$ for some $\kappa >0$ such that $(-\,\kappa + 3 C_A^2) \le 0$, then the assumption ${{\mathrm{inj}}}^M(x) < \frac{1}{4}{{\mathrm{inj}}}^Y(p)$ is already sufficient for Proposition 1.4 to hold, as *M*, as well as *Y*, do not have any conjugate points. In particular, the injectivity radius at some point at *M* or *Y* equals half of the length of the shortest geodesic loop based at that point.

## Characterization of Codimension One Collapse

In this section, we prove Theorem 1.1 and discuss the properties of the subspace of $\mathcal {M}(n,D)$ consisting of manifolds satisfying $C \le \frac{{{\mathrm{vol}}}(M)}{{{\mathrm{inj}}}(M)}$ for a chosen positive constant *C*.

We first observe that in the case of a noncollapsing sequence the statement of Theorem 1.1 is obviously true, as the limit space is a closed Riemannian manifold of the same dimension. Thus, we only consider the case of collapsing sequences in $\mathcal {M}(n,D)$.

The strategy of the proof of Theorem 1.1 is to first reduce the statement to sequences of sufficiently collapsed manifolds with invariant metrics in the sense of [2]. Then we prove Theorem 1.1 in that special case.

Thus, we first show that, for a collapsing sequence $(M_i, g_i)_{i \in \mathbb {N}}$, we can switch to invariant metrics $\tilde{g}_i$ without affecting the statement of Theorem 1.1.

### Lemma 4.1

Let $(M_i,g_i)_{i \in \mathbb {N}}$ be a collapsing sequence in $\mathcal {M}(n,D)$ with limit space *Y*. For $\delta >0$ sufficiently small and all $i \in \mathbb {N}$ sufficiently large, there is an invariant metric $\tilde{g}_i$ such that

$$\begin{aligned} \vert g_i - \tilde{g}_i \vert&< (e^{\delta }-1) + C(n,\delta ) d_{\text {GH}}(M_i, Y), \\ \vert \nabla _i - \tilde{\nabla }_i \vert&\le \delta + C_1(n, \delta ) d_{\text {GH}}(M_i, Y),\\ \vert \tilde{\nabla }^j_i \tilde{R}_i \vert&\le C(j, n, \delta )(1 + d_{\text {GH}}(M_i,Y)). \end{aligned}$$

In particular,

$$\begin{aligned}&e^{- \tau (d_{\text {GH}}(M_i, Y) \vert n, \delta )- \tau (\delta \vert n)} \frac{{{\mathrm{vol}}}\big (\tilde{B}^{M_i}_r(x)\big )}{\widetilde{{{\mathrm{inj}}}}^{M_i}(x)} \le \frac{{{\mathrm{vol}}}\big (B_r^{M_i}\big )(x)}{{{\mathrm{inj}}}^{M_i}(x)}\\&\quad \le e^{\tau (d_{\text {GH}}(M_i, Y) \vert n, \delta ) + \tau (\delta \vert n)} \frac{{{\mathrm{vol}}}\big (\tilde{B}^{M_i}_r(x)\big )}{\widetilde{{{\mathrm{inj}}}}^{M_i}(x)}, \end{aligned}$$

where $\tilde{B}^{M_i}_r(x)$ and $\widetilde{{{\mathrm{inj}}}}^{M_i}(x)$ are taken with respect to the metric $\tilde{g}_i$.

### Proof

First, we apply Theorem 2.2 with $\delta$ to the sequence and obtain the sequence $(M_i, \hat{g}_i)_{i \in \mathbb {N}}$ consisting only of *A*-regular manifolds, with $(A_j(n, \delta ))_{j \in \mathbb {N}}$. Furthermore, by choosing $\delta$ sufficiently small, Proposition 2.3 implies that $\vert \widehat{\sec }^{M_i} \vert \le (1 + c(n) \delta )$.

It follows with the estimates for the metrics $g_i$ and $\hat{g}_i$ in Theorem 2.2 that

4.1 $$\begin{aligned} e^{- \tau (\delta \vert n)} \frac{{{\mathrm{vol}}}\big (\hat{B}^{M_i}_r(x)\big )}{\widehat{{{\mathrm{inj}}}}^{M_i}(x)} \le \frac{{{\mathrm{vol}}}\big (B_r^{M_i}\big )(x)}{{{\mathrm{inj}}}^{M_i}(x)} \le e^{\tau (\delta \vert n)} \frac{{{\mathrm{vol}}}\big (\hat{B}^{M_i}_r(x)\big )}{\widehat{{{\mathrm{inj}}}}^{M_i}(x)} \end{aligned}$$

holds for sufficiently large $i \in \mathbb {N}$. Here, sufficiently large means that ${{\mathrm{inj}}}^{M_i}(x)$, resp. $\widehat{{{\mathrm{inj}}}}^{M_i}(x)$, is smaller than the conjugate radius of $(M_i, g_i)$, resp. $(M_i, \hat{g}_i)$, which is uniformly bounded in terms of the upper sectional curvature bound. The bound on the conjugate radius for $(M_i, \hat{g}_i)$ only changes slightly if choosing $\delta >0$ sufficiently small.

Next, we apply Theorem 2.4 to each element of $(M_i, \hat{g}_i)_{i \in \mathbb {N}}$ that satisfies $d_{\text {GH}}(M_i, Y) \le \varepsilon (n,D)$ to obtain an invariant metric $\tilde{g}_i$. Recall that $\varepsilon (n,D)$ is the constant from Theorem 2.1. This leads to a new sequence $(M_i, \tilde{g}_i)_{i \in \mathbb {N}}$. The claimed bounds on $\tilde{g_i}$ follow by combining the inequalities given in Theorem 2.2 and Theorem 2.4. In particular, after a small rescaling, $(M_i, \tilde{g}_i)_{i \in \mathbb {N}}$ lies again in $\mathcal {M}(n,D)$.

Furthermore, as $\vert \hat{g}_i - \tilde{g}_i \vert _{C^{\infty }} \le \tau (d_{\text {GH}}(M_i,Y) \vert n, \delta )$, it follows that

4.2 $$\begin{aligned} e^{- \tau (d_{\text {GH}}(M_i, Y) \vert n, \delta )} \frac{{{\mathrm{vol}}}\big (\tilde{B}^{M_i}_r(x)\big )}{\widetilde{{{\mathrm{inj}}}}^{M_i}(x)} \le \frac{{{\mathrm{vol}}}\big (\hat{B}_r^{M_i}\big )(x)}{\widehat{{{\mathrm{inj}}}}^{M_i}(x)} \le e^{\tau (d_{\text {GH}}(M_i, Y) \vert n, \delta )} \frac{{{\mathrm{vol}}}\big (\tilde{B}^{M_i}_r(x)\big )}{\widetilde{{{\mathrm{inj}}}}^{M_i}(x)}, \end{aligned}$$

uniformly for *i* sufficiently large, as before.

Observe that the sequences $(M_i, \hat{g}_i)_{i \in \mathbb {N}}$ and $(M_i, \tilde{g}_i)_{i \in \mathbb {N}}$ converge to the same limit space $\hat{Y}$. Furthermore, as $d_{\text {GH}}((M_i, g_i), (M_i, \tilde{g}_i) )$ is small, it follows by [7, Lemma 2.3] that the Lipschitz-distance between *Y* and $\hat{Y}$ is also small. In particular, *Y* and $\hat{Y}$ are homeomorphic and thus have the same Hausdorff dimension. Together with (4.1) and (4.2) the claim follows. $\square$

In order to prove Theorem 1.1 we consider the following *simplified setting*:

Let $(M_i, g_i)_{i \in \mathbb {N}}$ be a sequence in $\mathcal {M}(n,D)$ converging to a compact metric space *Y* of lower dimension. There is a large index *I* such that $d_{\text {GH}}(M_i, g_i) \le \varepsilon (n,D)$, where $\varepsilon (n,D)$ is the constant from Theorem 2.1, and ${{\mathrm{inj}}}^{M_i}(x) < \pi$ for all $x \in M_i$, $i \ge I$ . To prove Theorem 1.1, it is sufficient to consider such sequences $(M_i, g_i)_{i \ge I}$, where we can assume without loss of generality that $g_i$ is an invariant metric such that $(M_i, g_i)$ is $A(n,D, \delta )$-regular for all *i*, by Lemma 4.1. Here, we used that $d_{\text {GH}}(M_i,Y)$ is bounded from above by $\varepsilon (n,D)$, for all *i*.

The next proposition together with Lemma 4.1 proves the implication (i) to (ii) in Theorem 1.1.

### Proposition 4.2

Let $(M_i, g_i)_{i \in \mathbb {N}}$ be a collapsing sequence of *A*-regular manifolds in $\mathcal {M}(n,D)$ converging to a compact metric space *Y* in the Gromov–Hausdorff topology. Suppose that for each $i \in \mathbb {N}$, $d_{\text {GH}}(M_i,Y) \le \varepsilon (n,D)$, and ${{\mathrm{inj}}}^{M_i}(x) < \pi$ for all $x \in M_i$, and that the metric $g_i$ is invariant for the corresponding *N*-structure on $M_i .$

If $\dim _{\mathrm {Haus}}(Y) = (n-1)$, then for each $r>0$ there is a positive constant $C :=C(n,r,Y)$ such that

4.3 $$\begin{aligned} C \le \frac{{{\mathrm{vol}}}(B_r^{M_i}(x))}{{{\mathrm{inj}}}^{M_i}(x)} \end{aligned}$$

for all $x \in M_i$ and $i \in \mathbb {N}.$

### Proof

Since $\dim _{\mathrm {Haus}}(Y)= (n-1)$, it follows by [9, Proposition 11.5] that *Y* is a compact Riemannian orbifold. As the fibration, $\tilde{\eta }_i: FM_i \rightarrow \tilde{Y}$ defines an $\mathbb {S}^1$-bundle, it descends to an $\mathbb {S}^1$-orbifold bundle $\eta _i : M_i \rightarrow Y$.

Fix some $r > 0$. As $i \rightarrow \infty$, the ball $B^{M_i}_r(x)$ more and more resembles $\eta _i^{-1}(B^Y_r(\eta _i(x)))$. Thus, there is an index *I* such that for any $i > I$

$$\begin{aligned} \eta _i^{-1}(B^Y_{\frac{r}{2}}(p) ) \subset B^{M_i}_r(x) \end{aligned}$$

for all $p \in Y$ and $x \in F^i_p :=\eta ^{-1}_i (p)$ (e.g., one can use Toponogov’s theorem as the sequence lies in $\mathcal {M}(n,D)$).

Since the *T*-tensor of the Riemannian submersions $\tilde{\eta }_i$ is uniformly bounded by a constant $C_T(n,A)$, see Theorem 2.4, it follows, by considering the commuting diagram (2.1), that for any $r > 0$ there is a positive constant $C_1 :=C_1(r, n , C_T )$ such that, for all $i > I$,

$$\begin{aligned} {{\mathrm{vol}}}(B^{M_i}_r(x)) \ge C_1 {{\mathrm{vol}}}(B^Y_{\frac{r}{2}}(p)) {{\mathrm{vol}}}(F^i_p) = C_1 {{\mathrm{vol}}}(B^Y_{\frac{r}{2}}(p)) \, 2 {{\mathrm{inj}}}(F_p). \end{aligned}$$

The last equality holds as $F^i_p \cong \mathbb {S}^1$. Therefore,

$$\begin{aligned} \frac{{{\mathrm{vol}}}\left( B^{M_i}_{r}(x)\right) }{{{\mathrm{inj}}}^{M_i}(x)}&\ge C_1 {{\mathrm{vol}}}\left( B^Y_{\frac{r}{2}}(p)\right) \\&\ge 2 C_1 \inf _{i \in \mathbb {N}} \min _{p \in Y} {{\mathrm{vol}}}\Big (B^Y_{\frac{r}{2}}(p)\Big ). \end{aligned}$$

As *Y* is a smooth compact Riemannian orbifold, the above constant *C* is strictly positive. $\square$

To finish the proof of Theorem 1.1 it remains to show that (iii) implies (i). We again assume, without loss of generality, the same simplified setting as explained above. The main idea of the proof is to derive a contradiction by constructing an upper bound that converges to 0. This is done in the next proposition which, together with Lemma 4.1, finishes the proof of Theorem 1.1.

### Proposition 4.3

Let $(M_i, g_i)_{i \in \mathbb {N}}$ be a collapsing sequence of *A*-regular manifolds in $\mathcal {M}(n,D)$ converging to a compact metric space *Y* in the Gromov–Hausdorff topology. Suppose that for each $i \in \mathbb {N}$, $d_{\text {GH}}(M_i,Y) \le \varepsilon (n,D)$, ${{\mathrm{inj}}}^{M_i}(x) < \pi$ for all $x \in M_i$, and that the metric $g_i$ is invariant for the corresponding *N*-structure on $M_i .$

Then $\dim _{\mathrm {Haus}}(Y) = (n-1),$ if there is some $r >0$ and $C>0$ such that

$$\begin{aligned} C \le \frac{{{\mathrm{vol}}}(B_r^{M_i}(x))}{{{\mathrm{inj}}}^{M_i}(x)} \end{aligned}$$

for all $x \in M$ and all $i \in \mathbb {N}$.

### Proof

Let $(M_i, g_i)_{i \in \mathbb {N}}$ be a collapsing sequence in $\mathcal {M}(n,D)$ such that the Gromov–Hausdorff limit *Y* has codimension $k \ge 2$. Furthermore, assume that there are positive numbers *r* and *C* such that (4.3) is satisfied for all points $x \in M_i$ and all $i \in \mathbb {N}$.

Naber and Tian proved in [15, Theorem 1.1] that the limit space *Y* is a Riemannian orbifold outside a closed set *S* of Hausdorff dimension $\dim _{\mathrm {Haus}}(S) \le \min \lbrace n-5, \dim Y - 3 \rbrace$. Hence, $\hat{Y} :=Y \smallsetminus S$ is a Riemannian orbifold.

By Theorem 2.4, it follows that the second fundamental form of $\tilde{\eta }_i: FM_i \rightarrow \tilde{Y}$ is uniformly bounded by a constant $\tilde{C}_T(n,A)$. Therefore, considering the commutative diagram (2.1), it follows that for any $r > 0$ there is a constant $C_1(r,n,\tilde{C}_T)$ such that

$$\begin{aligned} {{\mathrm{vol}}}\big (B^{M_i}_r(x)\big ) \le C_1 {{\mathrm{vol}}}\big (B^Y_{r}(\eta _i(x))\big ) {{\mathrm{vol}}}\big (F^i_{\eta _i(x)}\big ) \end{aligned}$$

for any $$x \in M_i$$, $$i \in \mathbb {N}$$.

Let $p \in \hat{Y}$ be a regular point and $x \in F^i_p$. Then, there is some $\kappa >0$ such that $\overline{B^Y_{\kappa }(p)}$ is a compact Riemannian manifold with boundary.

Now, we consider the maps $\eta _i$ restricted to the preimage $\eta _i^{-1}\big ( \overline{B^Y_{\kappa }(p)} \big )$ for all *i*. These are Riemannian submersions between manifolds. Recall that the *T*-tensor of the Riemannian submersions $\tilde{\eta }_i$ is uniformly bounded in *i*, by Theorem 2.4. Hence, also the *T*-tensor of $\eta _i$ restricted to the preimage of $\overline{B^Y_{\kappa }(p)}$ is uniformly bounded by a constant $C_T$.

As the sequence $(M_i,g_i)_{i \in \mathbb {N}}$ only consists of *A*-regular manifolds, we can extract a subsequence, denoted by $(M_i, g_i)_{i \in \mathbb {N}}$, such that the Riemannian metrics $(\tilde{\eta }_i)_{*}(g_i^F)$ on $\tilde{Y}$ converges in $C^{\infty }$. Here $g_i^F$ denotes the metric on the frame bundle $FM_i$ induced by $g_i$. Therefore, it follows that the metrics $(\eta _i)_{*}(g_i)$ converges in $C^{\infty }$ on $\overline{B^Y_{\kappa }(p)}$. In particular, the sectional curvature on $B^Y_{\kappa }(p)$ can be uniformly bounded in *i*. Hence, by Gray-O’Neill’s formula, the *A*-tensor is uniformly bounded in norm by a constant $C_A$ on $B^Y_{\kappa }(p)$.

Now, let $\gamma$ be the noncontractible geodesic loop based at $x \in F_p$ such that $l(\gamma ) = 2 {{\mathrm{inj}}}^{M_i}(x)$. By taking *i* sufficiently large, the assumptions of Proposition 1.4 are fulfilled and therefore,

4.4 $$\begin{aligned} {{\mathrm{inj}}}(F^i_p) \le (1 + \tau ({{\mathrm{inj}}}^{M_i}(x) \vert k, C_T, C_A)) {{\mathrm{inj}}}^{M_i}(x) =:C_2 {{\mathrm{inj}}}^{M_i}(x). \end{aligned}$$

It follows easily from Gray-O’Neill’s formula that the sectional curvature of $F_p^i$ is bounded from below by $-K^2$ for some positive constant $K :=K(C_T)$. Hence, we apply [13, Corollary 2.3.2] and (4.4) to conclude

$$\begin{aligned} C&\le \frac{{{\mathrm{vol}}}(B^{M_i}_r(x))}{{{\mathrm{inj}}}^{M_i}(x)} \\&\le \frac{C_1 {{\mathrm{vol}}}(B^Y_r(p)) {{\mathrm{vol}}}(F^i_p)}{{{\mathrm{inj}}}^{M_i}(x)} \\&\le \frac{C_1 {{\mathrm{vol}}}(B^Y_r(p))\bigg ( C_3(k) {{\mathrm{inj}}}(F^i_p) \left( \frac{\sinh ({{\mathrm{diam}}}(F^i_p) K )}{K} \right) ^{k-1} \bigg ) }{{{\mathrm{inj}}}^{M_i}(x)} \\&\le C_1 C_2 C_3 {{\mathrm{vol}}}(B^Y_r(p)) \left( \frac{\sinh ({{\mathrm{diam}}}(F^i_p) K )}{K} \right) ^{k-1}. \end{aligned}$$

Since $(M_i, g_i)_{i \in \mathbb {N}}$ is a collapsing sequence, $\lim _{i \rightarrow \infty } {{\mathrm{diam}}}(F^i_p) =0$. As $k \ge 2$ by assumption, it follows that

$$\begin{aligned} \lim _{i \rightarrow \infty } \left( \frac{\sinh ({{\mathrm{diam}}}(F^i_p) K )}{K} \right) ^{k-1} = 0. \end{aligned}$$

This implies in the limit $C \le 0$ which is a contradiction. $\square$

As a further explicit example, we consider the Hopf fibration $\mathbb {S}^1 \rightarrow \mathbb {S}^3 \rightarrow \mathbb {S}^2(\frac{1}{2})$. We compute the constant from Theorem 1.1 for this collapsing sequence explicitly.

### Example 4.4

(Hopf fibration) Consider the sequence $(\mathbb {S}^3_i, g_i)_{i \in \mathbb {N}} \in \mathcal {M}(4, \pi )$. Here $(\mathbb {S}^3_i, g_i)$ denotes the total space of the Hopf fibrations where the fibers are scaled such that they have diameter $\frac{\pi }{i}$. This defines a collapsing sequence converging to $\mathbb {S}^2(\frac{1}{2})$. The Riemannian submersions $\eta _i: \mathbb {S}^3 \rightarrow \mathbb {S}^2(\frac{1}{2})$ are all totally geodesic and the integrability tensors are uniformly bounded by two. Let $r = \pi$, then

$$\begin{aligned} {{\mathrm{vol}}}\big (\mathbb {S}^3_i\big ) = {{\mathrm{vol}}}\left( B_{\pi }^{\mathbb {S}^3_i}(x)\right) = {{\mathrm{vol}}}(F_p^i) {{\mathrm{vol}}}\left( B_{\frac{\pi }{2}}(p)\right) = \frac{2 \pi }{i} {{\mathrm{vol}}}\left( \mathbb {S}^2 \left( 1/2\right) \right) = \frac{2 \pi ^2}{i}. \end{aligned}$$

Thus, we have that

$$\begin{aligned} \frac{{{\mathrm{vol}}}\left( B_{\pi }^{\mathbb {S}^3_i}(x)\right) }{{{\mathrm{inj}}}^{\mathbb {S}^3_i}(x)} = \frac{\frac{2 \pi ^2}{i}}{\frac{\pi }{i}} = 2 \pi = 2 {{\mathrm{vol}}}(\mathbb {S}^2 \left( 1/2 \right) ), \end{aligned}$$

which bounds this quotient uniformly in *i*.

We conclude this paper, by examining the following subspace of $\mathcal {M}(n,D)$.

### Definition 4.5

For given positive numbers *n*, *D*, and *C*, we define $\mathcal {M}(n,D,C)$ as the space consisting of all isometry classes of closed Riemannian manifold (*M*, *g*) in $\mathcal {M}(n,D)$ satisfying

$$\begin{aligned} C \le \frac{{{\mathrm{vol}}}(M)}{{{\mathrm{inj}}}(M)}. \end{aligned}$$

By Theorem 1.1 the closure $\mathcal {C}\mathcal {M}(n,D,C)$ of $\mathcal {M}(n,D,C)$ with respect to the Gromov–Hausdorff distance only consists of Riemannian manifolds or Riemannian orbifolds. For simplicity we consider each limit of a sequence in $\mathcal {M}(n,D,C)$ as an orbifold and understand a manifold as a special case. Recall that a manifold is an orbifold where each point is regular.

At this point we want to recall the following proposition due to Fukaya, (c.f. [9, Proposition 11.5]):

### Proposition 4.6

(Fukaya) Let $(M_i, g_i)_{i \in \mathbb {N}}$ be a sequence in $\mathcal {M}(n,D)$ converging to a compact metric space *Y* of codimension one. Then the groups $G_p$ (defined in Theorem 2.1) are all finite. In other words, *Y* is a Riemannian orbifold.

In particular, it follows, by the definition of a Riemannian orbifold, that $\mathcal {C} \mathcal {M}(n,D,C)$ only consists of *smooth* elements. For the definition of smooth elements in the closure of $\mathcal {M}(n,D)$, we refer to [7, Definition 0.4]. This observation is important, as we want to use the following lemma due to Fukaya (c.f. [7, Lemma 7.8]).

### Lemma 4.7

Let $(M_i, x_i)_{i \in \mathbb {N}}$ be a sequence of pointed manifolds in the $d_{\text {GH}}$-closure of $\mathcal {M}(n,D)$ converging to a smooth element (*Y*, *p*). Suppose that the sectional curvature of $M_i$ at $x_i$ are unbounded. Then the dimension of the group $G_p$ is positive.

Combining this with Proposition 4.6 we conclude the following properties of $\mathcal {C}\mathcal {M}(n,D,C)$.

### Theorem 4.8

The space $\mathcal {M}(n,D,C)$ is precompact in the Gromov–Hausdorff topology. Thus, any sequence $(M_i, g_i)_{i \in \mathbb {N}}$ contains a subsequence that either converges to a Riemannian manifold of the same dimension in the $C^{1, \alpha }$-topology or collapses to a compact Riemannian orbifold (*Y*, *h*) of Hausdorff-dimension $(n-1)$ such that $(\eta _i)_{*} g_i$ converges to *h* in the $C^{1, \alpha }$-topology on *Y*. Furthermore, any element *Y* in $\mathcal {C}\mathcal {M}(n,D,C)$ with $\dim (Y) = (n-1)$ satisfies $\Vert \sec ^Y \Vert _{L^{\infty }} \le K$ and ${{\mathrm{vol}}}(Y) > v$ for positive constants *v* and *K* depending on *n*, *D*, and *C*.

### Proof

Let $(M_i, g_i)_{i \in \mathbb {N}}$ be a sequence in $\mathcal {M}(n,D,C)$. Then there exists by Gromov’s compactness result a $d_{\text {GH}}$-convergent subsequence, converging to a compact metric space *Y*.

If $\dim (Y) = n$, then the injectivity radius of the manifolds $M_i$ is uniformly bounded from below by a constant $i_0$. Thus, this sequence lies in $\mathcal {M}(n,D,i_0)$ and the claim follows as this space is precompact in the $C^{1, \alpha }$-topology.

If $\dim (Y) < n$, then $\dim (Y) = (n-1)$ by Theorem 1.1. Thus, *Y* is a Riemannian orbifold by Proposition 4.6. Applying the *O*(*n*)-equivariant version of Gromov’s compactness result, there is a subsequence $(M_i,g_i)_{i \in \mathbb {N}}$ such that $(\tilde{\eta }_i)_{*}g^F_i$ converges on $\tilde{Y}$ in the $C^{1, \alpha }$-topology to an *O*(*n*)-equivariant metric. Here $g^F_i$ denotes the metric on $FM_i$ induced by $g_i$. As *Y* is a Riemannian orbifold, we also obtain that $(\eta _i)_{*} g_i$ converges in $C^{1, \alpha }$ to a Riemannian metric *h* on *Y*. This proves the first part of the proposition.

For the second part, assume that there is a sequence $(Y_i, h_i)_{i \in \mathbb {N}}$ of $(n-1)$-dimensional Riemannian orbifolds in $\mathcal {C}\mathcal {M}(n,D,C)$ such that there is a sequence of points $p_i \in Y_i$ where the sectional curvatures are unbounded in *i*. As each element $Y_i$ can be reached by a collapsing sequence in $\mathcal {M}(n,D)$, there is a subsequence $(Y_i)_{i \in \mathbb {N}}$ converging to an element $Y_{\infty }$ in $\mathcal {C}\mathcal {M}(n,D,C)$ and a point $p_{\infty }$ with unbounded sectional curvature. By a diagonal sequence argument and Theorem 1.1, it follows that $Y_{\infty }$ is a Riemannian orbifold. As $\mathcal {C}\mathcal {M}(n,D,C)$ is a subset of the $d_{\text {GH}}$-closure of $\mathcal {M}(n,D)$, we can apply Lemma 4.7. It follows that the group $G_{p_{\infty }}$ has positive dimension. This is a contradiction, as the group $G_{p_{\infty }}$ has to be finite by Proposition 4.6. Thus, there exists a constant *K*(*n*, *D*, *C*) as claimed.

The volume bound also follows by contradiction. Assume that there exists a sequence $(Y_i, h_i)_{i \in \mathbb {N}}$ such that $\dim (Y_i) = (n-1)$ for all *i* and such that ${{\mathrm{vol}}}(Y_i)$ converges to 0 as *i* tends to infinity. In other words the sequence $(Y_i, h_i)_{i \in \mathbb {N}}$ collapses. By a diagonal sequence argument, we obtain a sequence $(M_j, g_j)_{j \in \mathbb {N}}$ in $\mathcal {M}(n,D,C)$ converging to a space of at least codimension two. This is a contradiction, by Theorem 1.1. $\square$

## References

[1] Abresch U (1988). Über das Glätten Riemannscher Metriken.

[2] Cheeger J, Fukaya K, Gromov M (1992). Nilpotent structures and invariant metrics on collapsed manifolds. J. Am. Math. Soc..

[3] Cheeger J, Gromov M (1986). Collapsing Riemannian manifolds while keeping their curvature bounded. I. J. Differential Geom..

[4] Cheeger J, Gromov M (1990). Collapsing Riemannian manifolds while keeping their curvature bounded. II. J. Differential Geom..

[5] Cheeger J (1970). Finiteness theorems for Riemannian manifolds. Am. J. Math..

[6] Fukaya K (1987). Collapsing Riemannian manifolds to ones of lower dimensions. J. Differential Geom..

[7] Fukaya K (1988). A boundary of the set of the Riemannian manifolds with bounded curvatures and diameters. J. Differential Geom..

[8] Fukaya K (1989). Collapsing Riemannian manifolds to ones with lower dimension. II. J. Math. Soc. Jpn..

[9] Fukaya, K.: Hausdorff convergence of Riemannian manifolds and its applications, Recent topics in differential and analytic geometry, Advances Studies in Pure Mathematics, vol. 18, Academic Press, Boston, MA, pp. 143–238 (1990)

[10] Gromov M (1978). Almost flat manifolds. J. Differential Geom..

[11] Gromov, M.: Structures métriques pour les variétés riemanniennes, Textes Mathématiques [Mathematical Texts], vol. 1, CEDIC, Paris, Edited by J. Lafontaine and P. Pansu. (1981)

[12] Guijarro L, Walschap G (2000). The metric projection onto the soul. Trans. Am. Math. Soc..

[13] Heintze E, Karcher H (1978). A general comparison theorem with applications to volume estimates for submanifolds. Ann. Sci. École Norm. Sup..

[14] Jost J (2005). Riemannian Geometry and Geometric Analysis.

[15] Naber, A., Tian, G.: Geometric structures of collapsing Riemannian manifolds I, Surveys in geometric analysis and relativity, Adv. Lect. Math. (ALM), vol. 20, Int. Press, Somerville, MA, pp. 439–466 (2011)

[16] Peters S (1987). Convergence of Riemannian manifolds. Compositio Math..

[17] Rong X (1996). On the fundamental groups of manifolds of positive sectional curvature. Ann. Math..

[18] Shi W-X (1989). Deforming the metric on complete Riemannian manifolds. J. Differential Geom..

[19] Tapp K (2000). Bounded Riemannian submersions. Indiana Univ. Math. J..

